# Computed tomography provides enhanced techniques for longitudinal monitoring of progressive intracranial volume loss associated with regional neurodegeneration in ovine neuronal ceroid lipofuscinoses

**DOI:** 10.1002/brb3.1096

**Published:** 2018-08-23

**Authors:** Katharina N. Russell, Nadia L. Mitchell, Nigel G. Anderson, Craig R. Bunt, Martin P. Wellby, Tracy R. Melzer, Graham K. Barrell, David N. Palmer

**Affiliations:** ^1^ Faculty of Agriculture and Life Sciences Lincoln University Lincoln New Zealand; ^2^ Department of Radiology University of Otago Christchurch New Zealand; ^3^ Department of Medicine University of Otago Christchurch New Zealand; ^4^ New Zealand Brain Research Institute Christchurch New Zealand

**Keywords:** 3D reconstruction, Batten disease, brain, cranial ossification, CT, Hounsfield units, in vivo, longitudinal monitoring, NCL, neurodegeneration, neuroimaging, neuronal ceroid lipofuscinoses, radio‐density

## Abstract

**Introduction:**

The neuronal ceroid lipofuscinoses (NCLs; Batten disease) are a group of fatal neurodegenerative lysosomal storage diseases of children caused by various mutations in a range of genes. Forms associated with mutations in two of these, *CLN5 *and *CLN6, *are being investigated in well‐established sheep models. Brain atrophy leading to psychomotor degeneration is among the defining features, as is regional progressive ossification of the inner cranium. Ongoing viral‐mediated gene therapy trials in these sheep are yielding encouraging results. In vivo assessment of brain atrophy is integral to the longitudinal monitoring of individual animals and provides robust data for translation to treatments for humans.

**Methods:**

Computed tomography (CT)‐based three‐dimensional reconstruction of the intracranial volume (ICV) over time reflects the progression of cortical brain atrophy, verifying the use of ICV measurements as a surrogate measure for brain size in ovine NCL.

**Results:**

ICVs of NCL‐affected sheep increase for the first few months, but then decline progressively between 5 and 13 months in *CLN5^−/−^* sheep and 11–15 months in *CLN6^−/−^*sheep. Cerebral ventricular volumes are also increased in affected animals. To facilitate ICV measures, the radiodensities of ovine brain tissue and cerebrospinal fluid were identified. Ovine brain tissue exhibited a Hounsfield unit (HU) range of (24; 56) and cerebrospinal fluid a HU range of (−12; 23).

**Conclusions:**

Computed tomography scanning and reconstruction verify that brain atrophy ovine CLN5 NCL originates in the occipital lobes with subsequent propagation throughout the whole cortex and these regional differences are reflected in the ICV loss.

## 1 INTRODUCTION

The neuronal ceroid lipofuscinoses (NCLs, Batten disease) are a group of mainly autosomal recessively inherited lysosomal storage diseases caused by mutations in a number of genes that lead to degenerative and fatal encephalopathies in children (Haltia, 2006). Affected children progressively develop blindness, motor degeneration, epilepsy, and dementia, eventually reaching total dependency and premature death (Kousi, Lehesjoki, & Mole, 2012; Mole, Williams, & Goebel, 2005, 2011). There are no effective treatments. The NCLs are unified through severe cerebral neurodegeneration, neuroinflammation, and the nearly ubiquitous intracellular accumulation of storage bodies containing specific proteins (Kousi et al., 2012; Mole, Williams, & Goebel, 2005; Palmer, 2015; Palmer et al., 2015). A flock of sheep with a mutation in the *CLN5* gene and two with mutations in the *CLN6* gene provide well‐established large animal models that largely mirror the human pathological changes including the intracellular accumulation of subunit c of the mitochondrial ATP synthase (Cook et al., 2002; Frugier et al., 2008; Jolly et al., 1989; Jolly, Arthur, Kay, & Palmer, 2002; Palmer, 2015). Other common features of the ovine and human NCLs include progressive cerebral atrophy and enlargement of the cerebral ventricles (Frugier et al., 2008; Jolly et al., 2002, 1989 ; Woods et al., 1993). Neuroimaging techniques, such as magnetic resonance imaging (MRI) and computed tomography (CT), have been used to monitor changes of brain size and intracerebroventricular volumes in both human and ovine NCLs (Dyke et al., 2016; Jadav et al., 2014; Lobel et al., 2013; Sawiak et al., 2015; Valavanis, Friede, Schubinger, & Hayek, 1980; Woods et al., 1993). Viral‐mediated gene therapy trials are being conducted in affected sheep (Palmer, 2015). *In vivo* assessment of brain atrophy is integral to the longitudinal monitoring of individual animals and provides robust data for translation to human clinical trials.

It has been observed at *postmortem* examinations of NCL‐affected animals that their skulls are consistently thicker than those of controls and that the intracranial volume (ICV) reduces with disease development, suggesting that CT‐based measurement of the ICV could be a suitable surrogate measure for brain size changes in ovine NCL. Determination of tissue volumes from CT data requires discrimination of tissue boundaries. Tissues exhibit differences in their ability to attenuate radiation; that is, they have differing radio‐densities. The CT number or Hounsfield unit (HU) provides quantification of the extent to which a tissue attenuates X‐rays (Table 1) (Hounsfield, 1980) and can be used to identify boundaries between adjacent tissues. Human brain HU values range between 20 and 50, and those of cerebrospinal fluid (CSF) vary between −5 and 20 (Arimitsu, Di Chiro, Brooks, & Smith, 1977; Hacker & Artmann, 1978). Neurodegeneration may also alter the radio‐density of brain tissue, as has been reported in sheep and dog models of NCLs (Armstrong, Quisling, Webb, & Koppang, 1983; Woods et al., 1993). The aims of this study were to determine sheep‐specific HU thresholds for brain tissue and CSF, to investigate the relation of ICV and brain volume in NCL‐affected animals and controls and to perform a longitudinal study of ICV and brain volumes in CLN5‐ and CLN6‐affected sheep and controls.

**Table 1:** Radio‐density of different body materials (Modified from Hounsfield, 1980)

Hounsfield Units	Material
−1,000	Air
−90 to −70	Adipose
0	Water
20 to 60	Tissue (other than adipose and bone)
>500	Bone

## 2 MATERIALS AND METHODS

All experiments were performed in accordance with the Lincoln University Code of Ethical Conduct for the Use of Animals, under the New Zealand Animal Welfare Act 1999 and were approved by the Lincoln University Animal Ethics Committee. Computed tomography was conducted using a GE Prospeed CT scanner (GE Healthcare, Hyogo, Japan, 1997).

Previously performed CT scans of 56 sheep were analyzed to determine sheep‐specific HU thresholds for brain tissue and CSF. The sheep included both males and females aged between 3.6 and 68.4 months and were *CLN5^+/-^* and *CLN5* ^−^ *^/^* ^−^ Borderdales, *CLN6^+/-^* *CLN6* ^−^ *^/^* ^−^ South Hampshires and unrelated control Coopworth and Romney crosses. HU values of brain (average of grey and white matter) and CSF‐filled spaces were determined using ImageJ (Rasband, ImageJ, US National Institutes of Health, Bethesda, MD, USA) in five images selected from each image set based on the image quality and the visibility of the lateral cerebral ventricles. The average HU values of brain tissue were recorded from two 1‐cm^2 ^circular areas selected for their visibility and uniformity of tissue type within the area, and both cortical and subcortical areas were included. Likewise, a 1‐cm^2^ circular area was selected within each of the lateral cerebral ventricles and the average HU values of these were recorded. Following this, the 99.9% confidence intervals representing the range of HU values of brain tissue and CSF spaces were determined.

**Figure 1:**
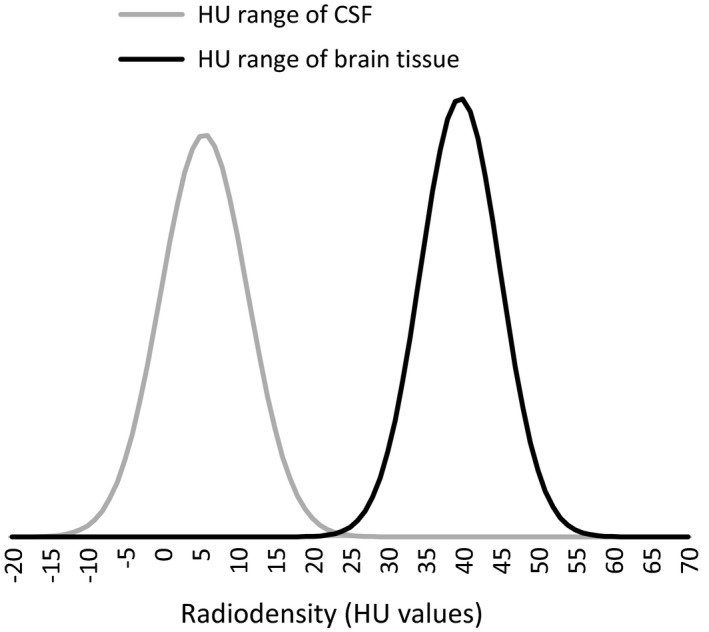
Range of radio‐density representing ovine brain tissue and CSF. Normal distribution of the Hounsfield units (HU) representing the radio‐densities of ovine brain tissue (HU range of brain tissue) and those of ovine cerebrospinal fluid (HU range of CSF) (*n* = 72). Mean HU of brain tissue = 39.7; *SD* = 5.3; 99.9% confidence interval [24; 56]. Mean HU of CSF = 5.6; *SD* = 5.8; 99.9% confidence interval [−12; 23]. [Correction added on 04 September 2018, after first online publication: mean HU and SD values have been corrected.]

For the longitudinal investigations and the comparison of ICV and brain size, scan settings were as follows: helical, 120 kV, 100 mA, 1 mm slice thickness at a 1‐mm slice interval and image analyses were performed in 3D Slicer 4.3.1 freeware (https://www.slicer.org; 3D Slicer: http://scicrunch.org/resolver/SCR_005619) (Fedorov et al., 2012). The ICVs were measured from the first image containing the olfactory bulbs to the final image containing cerebellum using the previously established HU threshold for both brain tissue and CSF spaces, ‐12; 56 (see Section 3, Figure 1) using the “Editor” tool. The volumes of the third and lateral cerebral ventricles were measured using the previously established CSF HU threshold (−12; 23). The remaining CSF‐filled spaces (the fourth cerebral ventricle, cerebellar aqueduct, and subarachnoid spaces) were not included in the CSF space measurements because the resolution of the CT scans was insufficient for accurate discrimination between brain tissue and CSF in these regions. Threshold‐based selection of the areas was followed by manual correction for beam hardening and other scanning artefacts. The “MakeModelEffect” tool was used to produce three‐dimensional (3D) reconstruction and volumetric measurements of the regions of interest.

The relationship between ICV and brain volume was examined using severed heads from nine NCL‐affected male, two NCL‐affected female, and 14 unaffected female sheep aged 8–69 months that were euthanized for other reasons than this study. Following exsanguination, the heads were CT scanned and the ICV and lateral cerebroventricular volumes measured using the protocols described above. Following CT scanning, the skulls and meninges were opened dorsally and the brains removed, leaving the pituitary glands, olfactory bulbs, and the dura mater behind. The spinal cords were severed transversely at a point perpendicular to the caudal margin of the cerebellum. Within four hours of collection, brain volumes were measured using a water displacement method. A horizontal line was drawn on the outside of a glass beaker, which was then filled with water at room temperature to this line. Next a brain was placed in the beaker, which caused the water level to rise as the brain sank. Water was removed until the water level was back to the initial line and the volume removed was recorded. The ratios of ICV to brain volume were calculated for both affected and normal sheep. The specific gravity of each brain was calculated by dividing the fresh brain weight by the water displacement volume, and the effects of disease status on ICV, brain‐specific gravity, and ratio of ICV to brain volume were investigated.

All observations indicate that the neuropathology in ovine CLN5 Batten disease is independent of gender and sheep of all genders (rams, ewes, and wethers) were used. The longitudinal investigations included a total of 24 animals, 12 animals born in 2014 (six affected males, six female controls), and 12 born in 2015 (six affected females, six female controls). Each group of 12 contained *CLN6* ^−^ *^/^* ^−^ (*n* = 3) and *CLN6^+/-^*(*n* = 3) South Hampshire sheep and *CLN5* ^−/−^ (*n* = 3) and *CLN5^+/-^*(*n* = 3) Borderdale sheep. All heterozygous sheep (i.e., *CLN5^+/-^* and *CLN6^+/-^*) were clinically normal and served as control animals. The sheep were aged between 3 and 5.8 months when first scanned and scans were performed bimonthly until an age of 15–19 months, depending on the clinical condition of individuals. They were kept outdoors on pasture. Prior to each CT scan, the sheep were fasted for 18–24 hr and then weighed. On the day of CT scanning, the sheep were anaesthetized by intravenous injection of a mixture of 0.8 mg/kg live weight (LW) diazepam (Pamlin injection, Troy Laboratories NZ Pty Ltd, Auckland, New Zealand) and 17 mg/kg LW of ketamine hydrochloride (Phoenix Ketamine injection, Phoenix Pharm Distributors Ltd, Auckland, New Zealand). They were then placed on a wooden stretcher in dorsal recumbency, and the stretcher was loaded onto the CT scanner. The heads were scanned following the protocol described above. After scanning, the sheep were taken to a padded wake‐up area and remained under observation until they recovered, when they were returned to their corresponding flocks.

For statistical analysis of the longitudinal data, animals were grouped by breed and genotype (*CLN5^+/-^, CLN5* ^−/−^ *, CLN6^+/-^, CLN6* ^−^ *^/^* ^−^) and the repeated measurements were allocated into age‐groups (3, 5, 7, 9, 11, 13, 15, 17, and 19 months). Means and corresponding standard errors of the mean (*SEM*) were calculated for ICV and ventricular volumes at each age for each group of animals. The change in ICV was calculated for each animal for each period (3–5 months, 5–7 months, etc.), and the mean accumulative ICV gain or loss (from 3 to 19 months) was determined for each genotype. Data processing and statistical analyses were performed in Microsoft Excel 2016 (Microsoft Corp., Seattle, WA, USA) and GenStat for Windows 16th Edition (http://scicrunch.org/resolver/SCR_014595, VSN International 2011, Hemel Hempstead, UK). Student's *t* tests were performed for pairwise comparisons of affected and normal (control) animals. Repeated ANOVAs were performed on longitudinal data. Results are reported as means ± *SEM*. Differences are regarded significant where *p* < 0.05.

## 3 RESULTS

The mean brain tissue HU value including both affected and unaffected animals after exsanguination was 39.5 with a standard deviation of 5.2, and thus, 99.9% of the ovine brain tissue was represented by the HU interval (24; 56). Likewise, 99.9% of CSF spaces was represented in the HU value interval of (−12; 23; Figure 1). The mean radio‐density (HU value) of brain tissue in NCL‐affected animals (*n* = 42) was greater than that of brain tissue from unaffected controls (*n* = 14) (mean affected = 40.3 ± 0.3; mean control = 38.4 ± 0.8; *p* = 0.037). There was no such difference in the radio‐density of CSF‐filled spaces (mean affected = 5.6 ± 0.5; mean control = 5.5 ± 0.6; *p = *0.8).

Brain volumes measured after *post mortem* by water displacement, and the corresponding ICVs measured from CT scans were both lower for NCL‐affected animals than those of controls (Table 2). Both CT scan ICVs and brain volumes of unaffected control animals closely matched, but the ICVs of NCL‐affected animals measured by CT scanning were approximately 4 ml larger than the corresponding brain volumes measured by displacement. Based on this, the ratio of ICV to brain volume was larger in NCL‐affected animals compared with unaffected controls. Disease status had no effect on the specific gravity of the brain (Table 2).

**Table 2:** Brain and intracranial volume comparisons

Measurement	Affected (mean ± *SEM*)	Control (mean ± *SEM*)	*p*
ICV (ml)	80.5 (± 2.3)	99.9 (± 1.7)	*<*0.001
Volume (ml)	76.5 (± 2.1)	101.1 (± 1.7)	*<*0.001
ICV:Volume ratio	1.06 (± 0.02)	0.99 (± 0.01)	0.03
Specific gravity of brain tissue (g/cm^3^)	0.98 (± 0.2)	1.01 (± 0.01)	0.11

Note

Volumetric brain measurements of NCL‐affected (*n* = 11) and unaffected control (*n* = 14) sheep. Intracranial volumes (ICV) measured on CT scans and brain volumes (Volume) measured by water displacement. *p* ≤ 0.05 regarded as significant.

**Figure 2:**
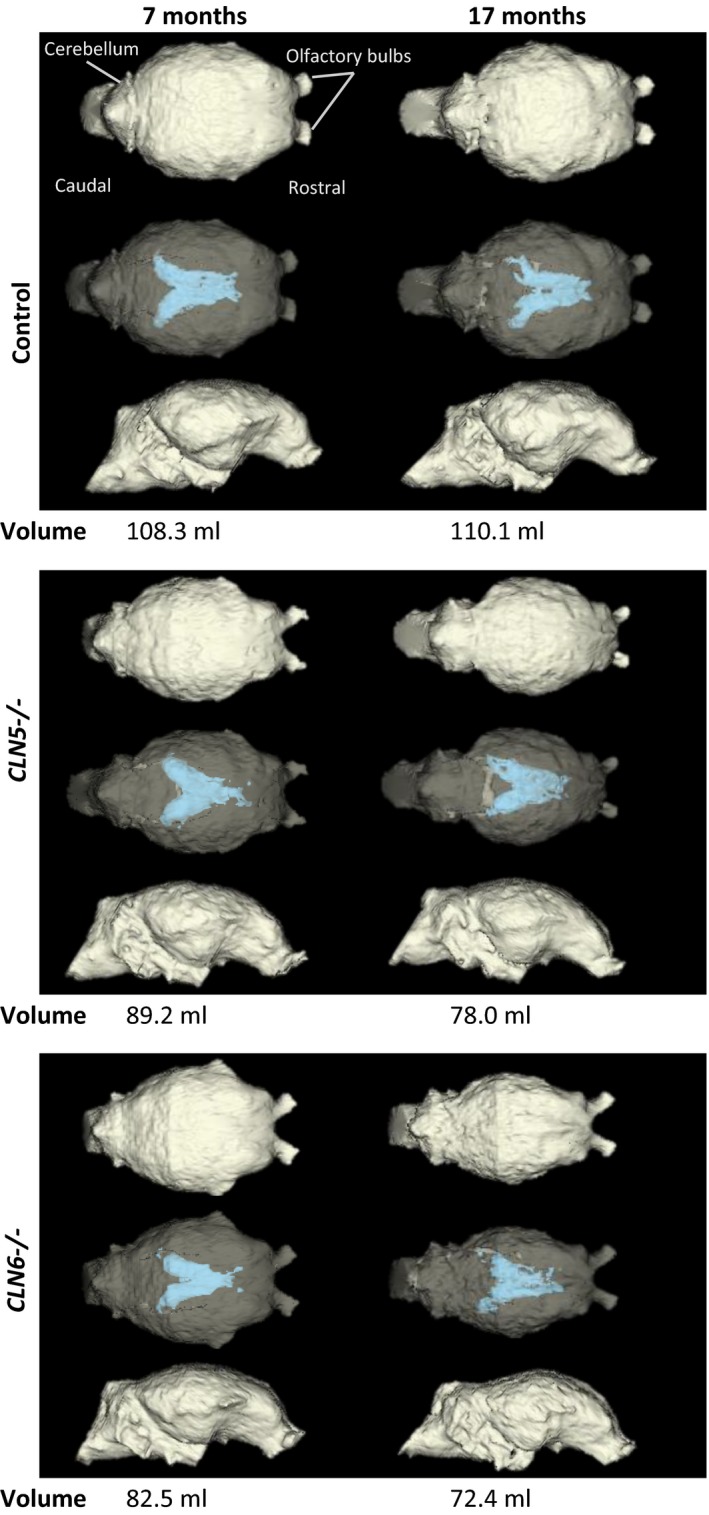
Representative 3D models of cranial volumes of a CLN5 and a CLN6 affected sheep and a control. Computer tomography (CT)‐based three‐dimensional (3D) modelling of intracranial volumes (ICV). In both *CLN5* ^−^ *^/^* ^−^ and *CLN6* ^−^ *^/^* ^−^, the loss of ICV and enlargement of lateral cerebral ventricles (blue) were evident at 7 months of age and were pronounced at 17 months compared with the healthy control. The top row of each block shows the dorsal view, the middle row shows the semi‐opaque dorsal view so that the lateral ventricles are visible, and the bottom row shows the lateral view

The CT‐based 3D reconstructions of the ICV of both *CLN5* ^−/−^ and *CLN6* ^−/−^ animals showed obvious visible changes compared with controls (Figure 2). By 7 months of age, the affected ICVs of both affected genotypes were smaller than those of age‐matched controls. At 17 months, the regionality of the brain atrophy was reflected in the shape of the ICVs. Whereas the cerebellar area of the ICVs was largely unchanged, the cerebral cortical areas appeared to be considerably thinner than those of controls, particularly in the occipital region (Figure 2, https://www.tinkercad.com/things/1dlXELC0gvp-sheep-brain-cavity-19mth-control, https://www.tinkercad.com/things/5Y1f9AKrEiA-sheep-brain-cavity-19mth-batten-disease). Overall, ICVs from 17‐month‐old affected animals of both genotypes were smaller than those of controls, and also smaller than that they were at 7 months of age.

**Figure 3:**
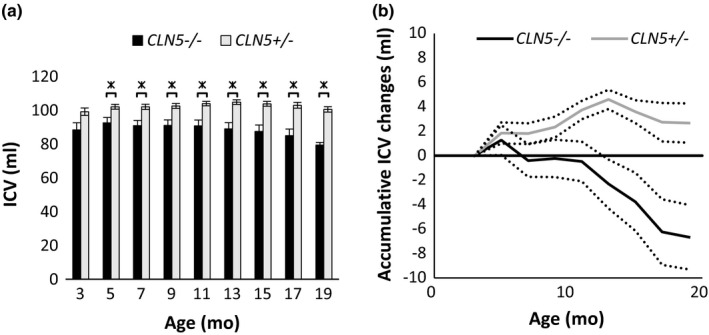
Development of intracranial volume (ICV) in CLN5 sheep. ICV development of* CLN5* ^−^ *^/^* ^−^ and *CLN5^+/-^* sheep between 3 and 19 months (mo). (a) Mean ICV ±SEM at different ages (* indicating significance at *p* ≤ 0.05). (b) Mean accumulative ICV gain or loss (± *SEM*, dotted lines) from 3 mo of age. *CLN5^+/-^*: 3 mo *n = *3; 5–17 mo *n* = 6; 19 mo *n* = 3. *CLN5* ^−^ *^/^* ^−^: 3 mo *n* = 3; 5–17 mo *n* = 6; 19 mo *n* = 2

The progression of changes in ICVs of Borderdale (*CLN5* ^−/−^ and *CLN5* ^+/‐^) sheep is summarized in Figure 3. By 3 months, the mean ICV of *CLN5* ^−/−^ sheep was smaller than that of unaffected age‐ and breed‐matched controls. This difference became statistically significant by 5 months and remained so until the end of the trial period at 19 months. The accumulative change of ICV in *CLN5^+/-^* control animals initially increased between 3 and 5 months, then remained steady until approximately 9 months, followed by another growth phase until leveling off from about 15 months of age. The mean ICV of *CLN5* ^−/−^ sheep initially increased between the ages of 3 and 5 months, similarly to that of controls. Thereafter, the volume decreased, plateaued between 7 and 11 months, and then decreased rapidly until the end of the trial period at 19 months. On average, *CLN5* ^−/− ^sheep (*n* = 6) lost a total ICV of 6.7 ± 2.6 ml between 3 and 17 months, whereas the mean ICV of control sheep (*n* = 6) increased by 2.7 ± 1.6 ml over that period.

**Figure 4:**
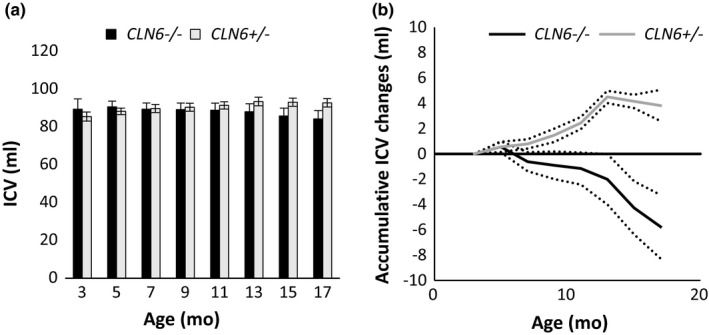
Development of intracranial volume (ICV) in CLN6 sheep. ICV development of* CLN6* ^−^ *^/^* ^−^ and *CLN6^+/-^* sheep between 3 and 19 months (mo). (a) Mean ICV ±SEM at different ages. (b) Mean accumulative ICV gain or loss (± *SEM*, dotted lines) from 3 mo of age. *CLN6^+/-^*: 3 mo *n = *3; 5–17 mo *n* = 6. *CLN6* ^−^ *^/^* ^−^: 3 mo *n* = 3; 5–17 mo *n* = 6

Changes in the ICVs of South Hampshire sheep (*CLN6* ^−/−^ and *CLN6^+/-^*) are summarized in Figure 4. The accumulative change of ICV in *CLN6^+/-^* control animals was similar to that of the* CLN5^+/-^* control animals. A slow phase of ICV gain occurred until the age of approximately 10 months, followed by a short steep increase in ICV until approximately 15 months of age, when the ICV plateaued. The accumulative change of ICV in *CLN6* ^−/−^ animals was similar to changes in the *CLN5* ^−^ *^/^* ^−^ animals. An initial increase was followed by a slight decline between 5 and 7 months, a plateau until approximately 10 months of age and then volumes declined rapidly until the end of the trial period at 17 months. A total mean ICV loss of 5.8 ± 2.5 ml occurred in *CLN6* ^−^ *^/^* ^− ^sheep (*n* = 6) between 3 and 17 months, while controls (*n* = 6) gained 3.8 ± 1.2 ml ICV on average over the same period.

**Figure 5:**
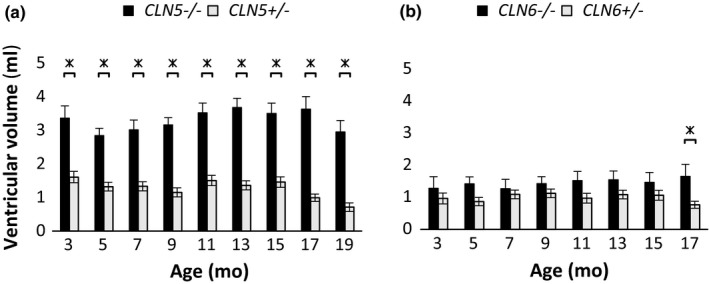
Cerebral ventricular volumes of CLN5 and CLN6 sheep. Volumes of intracerebral ventricles (lateral and third ventricles combined) of *CLN5* ^−^ *^/^* ^−^ and *CLN6* ^−^ *^/^* ^−^ animals and heterozygous controls at different ages (months, mo). (a) Mean ventricular volumes (± *SEM*) of* CLN5* ^−^ *^/^* ^−^ and *CLN5^+/-^* animals. (b) Mean ventricular volumes (± *SEM*) of* CLN6* ^−^ *^/^* ^−^ and *CLN6^+/-^* animals. Significance indicated with * where *p* ≤ 0.05, Student's *t* test. *CLN5^+/-^*: 3 mo *n = *3; 5–17 mo *n* = 6; 19 mo *n* = 3. *CLN5* ^−^ *^/^* ^−^: 3 mo *n* = 3; 5–17 mo *n* = 6; 19 mo *n* = 2. *CLN6^+/-^*: 3 mo *n = *3; 5–17 mo* n* = 6; *CLN6* ^−^ *^/^* ^−^: 3 mo *n* = 3; 5–17 mo *n* = 6

The mean volumes of the lateral and third cerebral ventricles are summarized in Figure 5. Those of *CLN5* ^−/−^ animals were significantly larger than those of controls throughout the entire trial period, and although the mean ventricular volumes of *CLN6* ^−/−^ animals tended to be higher than those of controls, this did not become statistically significant until 17 months of age.

## 4 DISCUSSION

Neuroimaging has been a component of disease monitoring in NCL research for some time but, until recently, the methods were basic and mainly focused on time‐point analyses rather than longitudinal monitoring of individuals. This study sets out to investigate the improvements that could be achieved through automated methods of image analysis to establish the relationship between the reduction in ICV and brain atrophy, and describe the patterns of ICV change in CLN5‐affected Borderdale, CLN6‐affected South Hampshire, and unaffected control sheep.

Measurements of radio‐densities of brain tissue and CSF spaces in NCL‐affected sheep and unaffected controls led to improved image analysis. An increased radio‐density of brain tissue, measured in HU, was observed in affected animals compared with controls. This has also been described in other models (ovine and canine) of NCL (Armstrong et al., 1983; Woods et al., 1993). The specific gravity of brains from affected animals was not increased even though the brains of affected sheep have been described as firmer in texture than those of controls (Jolly et al., 1989). The range of HU values representing brain tissue and CSF spaces reported here are in close agreement with findings from similar investigations in humans, where the mean HU value of grey matter was 39, that of white matter 32 and that of CSF ranged between −5 and 20 (Arimitsu et al., 1977; Hacker & Artmann, 1978). The present results have enabled semi‐automated analyses of CT scans to be used for longitudinal monitoring of treatment trials in NCL sheep and to create 3D images.

The comparison between CT‐based volumetric measurements of the cranial vault and brain volume measured by water displacement confirms the validity of the CT measurements as a surrogate for brain volume. The ratio of ICV to brain volume in normal sheep was close to unity. However, the ratio for NCL‐affected animals was 1.06, which is larger (*p = *0.03) than that of normal sheep and shows a slight discrepancy between the ICV and brain volume measurements recorded here. The reduction in ICV in NCL‐affected animals and the 3D reconstruction thereof presented in this study support the theory of an ongoing ossification of the skull in these animals. However, the larger ratio of ICV to brain volume indicates that the brain atrophy precedes this ossification, suggesting that the ossification is not causative of brain atrophy, but is rather a symptom of it. This is in agreement with an increase in space between the atrophied brain and the cranial vault of *CLN6* ^−^ *^/^* ^−^ NCL‐affected sheep reported earlier (Cook et al., 2002).

In human NCLs and other forms of dementia, remodeling of the skull is unusual and is mainly seen in diseases that also affect other parts of the skeleton, such as mucopolysaccharidosis and other skeletal dysplasias (Jelin et al., 2017). However, thickening of the cranium, also called hyperostosis cranii *ex vacuo*, has been described in hydrocephalic children sporadically, usually as a side effect of successful ventricular shunting, and as a rare consequence of brain atrophy (Di Preta, Powers, & Hicks, 1994; Wolf & Falsetti, 2001). More detailed investigations into the molecular mechanism, and the regional changes are needed to understand the dynamics of ICV changes in ovine NCL as well as in other species. The present ovine NCL models provide an excellent platform from which to investigate this phenomenon longitudinally.

Although it has been long established that CLN5‐ and CLN6‐affected sheep exhibit a reduction in brain size (Jolly & West, 1976; Jolly et al., 2002; Jolly, Janmaat, Graydon, & Clemett, 1982), longitudinal monitoring is required to describe the rate of this change. As brain size is related to overall body size (Courchesne et al., 2000; Ebinger, 1974; Edland et al., 2002), large individual variation would be expected in the measurements of ICV, and this might overshadow disease effects. Assessment of the rate of change instead of actual volumes (Figures 3 and 4) yields a better understanding of disease mechanisms by accounting for the confounding effects of body size and weight. This becomes particularly clear when comparing the mean ICV data of CLN5 Borderdale and CLN6 South Hampshire sheep, which are two breeds that differ in both build and size. In this study, *CLN5* ^−^ *^/^* ^−^ animals could easily be distinguished from *CLN5^+/-^* animals based on ICV at three months (Figure 3). This was not possible in CLN6 animals until the very end of the trial period, as the mean ICV of CLN6‐affected animals remained similar to that of controls for longer (Figure 4). However, the patterns of ICV change in different genotypes became obvious when analyzing the accumulative change instead of the mean volume. With this, it is possible to distinguish between affected animals and controls from a young age. For both CLN5 and CLN6 sheep, the present study showed that the ICV of affected animals changed only minimally until the approximate age of 10 months, which can be described as an absence of gain rather than an active loss, while the ICV of control animals steadily increased throughout the same period. After this, the ICV was reduced in both *CLN5* ^−^ *^/^* ^−^ and *CLN6* ^−^ *^/^* ^−^ animals, while the ICV of control animals plateaued. The 3D reconstructions revealed that the ICV changes follow the regionality of neuropathology that has been previously described (Oswald et al., 2005; Oswald, Palmer, Kay, Barwell, & Cooper, 2008).

The volumes of the third and lateral ventricles of all four genotypes were relatively stable throughout the entire study period. The third and lateral ventricular volumes of *CLN5* ^−^ *^/^* ^−^ animals were larger than those of controls and a lesser similar trend was observed in *CLN6* ^−^ *^/^* ^−^ animals, which did not become significant until the very end of the trial period (Figure 3). Overall, ventricular enlargement was more pronounced in *CLN5* ^−^ *^/^* ^−^ animals than in *CLN6* ^−^ *^/^* ^−^ animals, which may indicate a differing pattern of brain atrophy in the different types of ovine NCL. In recent past, a high‐resolution sheep brain MRI atlas has been established based on the brains of 18 sheep and it showed that the mean volume of the lateral and third ventricles combined was approximately 4.9 ml (Ella & Keller, 2015; Ella, Delgadillo, Chemineau, & Keller, 2017). The discrepancy between these measures and the measures recorded in the present study is likely consequent to differences in the measurement techniques and issues with the spatial resolution of the CT scanner used, and the present measures reflect the ventricular volumes sufficiently to compare affected and normal sheep within the same trial.

This study was limited by the age of the CT scanner available to the project, which has an inferior resolution compared with modern machines. The low resolution was insufficient to accurately discriminate tissue borders for small CSF‐filled spaces, leading to varying measures of the ventricular volumes in this study. Newer CT scanners would overcome this problem and enable more detailed measures of the CSF space, including the 4th ventricle and the subarachnoid space. The use of MRI instead of CT would increase the resolution and discrimination of soft tissue boundaries. However, the availability of MRI for large animal model research is limited by costs and lack of animal MRI facilities leading to logistical issues as well as longer scan times involving an increased need for anaesthesia. CT scanning, on the other hand, is much faster than MRI and in the present situation was easily available on‐site.

The present results show that CT‐based measurements of the intracranial volumes yield robust longitudinal information for the in vivo monitoring of ovine NCL. The findings of this study are significant as the techniques developed here for repeated, noninvasive, measures are already being used as valuable tools for the assessment of disease progression in trials of potential new therapies for the treatment of human NCL (Mitchell et al., 2018).

## CONFLICT OF INTEREST

The authors declare no conflict of interests.
